# Scientific Scoundrelism

**Published:** 1891-10-10

**Authors:** 


### The Doctor's Armchair.

### SCIENTIFIC SCOUNDRELISM.

"By these tests the Belladonna Plaster showed an equivalent of twenty per cent., Belladonna extract at a standard of two per cent. Atropia. Two other brands were tested, yielding less than half these amounts, and a decided colouring of lamp-black." A kind of rough and untutored simplicity is the prevailing note of savage life; a smooth and polished artificiality is the characteristic of the civilisation of the wealth-loving classes; a graceful and natural simplicity is the finished product of high culture and honourable character.

Success is held to be the justification of doubtful methods. A thief ceases to be a thief if he can but carry on his thieving outside the pale of the law. The man who puts a quart of water into a gallon of milk, and sells the mixture as the unadulterated produce of the cow, may be fined or sent to prison; but the man who makes his Belladonna plasters of less than half the strength required by the Pharmacopœia, and brings them up to the standard colour by the addition of lamp-black, can hold his head high among honourable men, and proudly fill the position of churchwarden or chapel deacon. He is a man of science. He knows exactly what to do in order to secure a first-rate price for a fourth-rate article, without incurring the risk of either fine or imprisonment.

The pump-loving milkman is a sorry creature, one of the meanest of the mean. If he were to sell pure milk he would get a profit of from twenty to twenty-five per cent. on all the milk sold; that is, twenty per cent, twice a day, all the year round, Sundays included. If this is not sufficient profit for any man with the minutest molecule of a conscience, what is? The milkman who calls in the aid of the pump is an ingrained and inveterate thief of a low, stupid, and degraded type. He ought never to be allowed the option of a fine. Seeing that he is a common thief, on what ground of reason can he be treated as a mere misdemeanant?

But there are scoundrels and scoundrels. It seems almost too much to believe that any man should put lampblack into Belladonna plaster; yet that is what is done by at least one, and probably a good many black-coated, high-collared, and kid-gloved knaves. According to a recent chemical assay, the results of the examination of four samples of Belladonna plaster were as follow: Plaster A contained 0·588; plaster B, 0·264; plaster C., 0·208; plaster D., 0·045. Now it is manifest that the people who supply remedies of these characters for the sick and suffering are not common thieves only—they are quite willing to be murderers as well, and murderers on the most extensive scale. For a man who will make a Belladonna plaster twenty times weaker than that plaster ought to be, and prescribing physicians believe it to be, will not be content with the adulteration of Belladonna plasters. He will adulterate his other drugs to the same tune wherever he possibly can. It may often happen that a remedy prescribed at its full and proper strength will save life, whilst the same remedy administered of not more than a twentieth part of the strength required by the patient's condition may result in death. Who is responsible for the fatal issue? The doctor who prescribed the remedy that would have saved the patient's life, or the scientific manipulator of chemicals who betrayed the physician by putting into his hands a weapon that broke to pieces in the mere handling?

The adulteration of remedies that are to be used for the sick stands on quite a different plane from the food adulteration question. The food adulterator often does very little harm. Of course, he is a thief if he does not properly label his adulterated wares. But the adulterator of drugs—What kind of work is his? He poisons or renders useless the medicines that are trusted by the physician and the helpless patient to relieve the anguish of pain, to lessen the tortures of fever, to soothe the almost insupportable distresses of the mind, to ward off impending death, to win back reluctant life; the remedies that are the hope of the bread-winner in the hour of his mortal peril, of the despairing mother as she thinks of her helpless children, and of the child that wrings the hearts of its parents by its piteous pleadings for help. The watchful physician and the tender wife or mother administer the potion that is to heal and save. But the medicine is a delusion: it is no better than water from the brook: and the man who made it or ordered it to be made knew that it was a delusion. Man! Do not call him a man: and do not degrade the devil by calling him a devil. Do not hang him, for that would contaminate the hangman. Cast him out into some outer darkness which is neither purgatory nor even the City of Dis, but black midnight, in which he shall forever dwell alone.

There seems to be no way of reaching the born adulterator, either by law or by public degradation. But he who believes, with Dante, in a future retribution, feels and sternly rejoices in the feeling, that the successful scoundrel will not escape for ever. The just man's consciousness insists upon the necessity for retribution: the exact man feels that all sorts of wrong must be counter-balanced sooner or later by right. These are primitive instincts, integral parts of the consciousness; and no amount of plausible special pleading can remove them so much as a hairs-breadth from the mind. The physician, whose work is a religion to him, experiences such an uprising of moral indignation when he thinks of the consequences of the drug adulterator's deeds, that he would willingly see the creature annihilated. What, indeed, is such a human soul worth except annihilation? The drug adulterator is quite willing that thousands of other human lives shall be extinguished for his profit. Let him reap what he is so willing to sow. Foulon, hanged with a wisp of grass in his mouth, is a historical figure that satisfies the primitive sentiments of justice. For our part we would like to see every drug adulterator reduced to the necessity of feeding entirely on his own drugs as long as they would keep him alive.

---

Dr. Weissblum reports in a German periodical the results of some experiments which he has made with aristol. With the exception of cases of eczema, it seems to have been anything but satisfactory. He employed as a vehicle either olive oil, vaseline, or lanolin.

